# Meowing dogs: can dogs recognize cats in a cross-modal violation of expectancy task (*Canis familiaris*)?

**DOI:** 10.1007/s10071-023-01783-0

**Published:** 2023-05-12

**Authors:** Miina Lõoke, Cécile Guérineau, Anna Broseghini, Lieta Marinelli, Paolo Mongillo

**Affiliations:** Dipartimento di Biomedicina Comparata e Alimentazione, University of Padua, Viale dell’Università 16, 35020 Legnaro, PD Italy

**Keywords:** Cross-modal, Dog, Expectancy violation, Species recognition, Videos

## Abstract

Dogs can recognize conspecifics in cross-modal audio–video presentations. In this paper, we aimed at exploring if such capability extends to the recognition of cats, and whether it is influenced by exposure to these animals. To reach our aim, we enrolled 64 pet dogs. Half of the dogs were currently living with cats, while the rest had never been living with cats, nor were at the time of the experiment. All dogs underwent a cross-modal violation of expectancy experiment, where they were presented with either a cat or a dog vocalization, followed by a video of either species on a blank background. The result revealed that dogs did not exhibit a surprise reaction towards the incoherent stimuli of a cat vocalization and a dog video or vice-versa, implying that they had not recognized the stimuli portraying cats. The pattern of results did not differ between dogs living or not with cats, implying that exposure to a limited number of cats, however, prolonged, is not sufficient to grant dogs with the ability to recognize them on audio–video presentations. We propose that the lack of recognition could be due to the small number of individual cats the dogs are regularly exposed to, or to the possible lack of early exposure to cats during the socialization phase.

**Supplementary Information:**

The online version contains supplementary material available at 10.1007/s10071-023-01783-0.

## Introduction

Animals often need to classify individuals into meaningful groups, such as conspecifics, preys or predators, to manifest appropriate behaviours (Gherardi et al. 2012). The ability to classify someone or something is generally described under the umbrella term of “recognition” (sensu, Wiley 2013). However, since in some cases this term has been used to refer exclusively to individual recognition, rather than class recognition, we feel compelled to specify that in this paper, we will use the term in its broader meaning.

Recognition is dependent on two interrelated factors: predisposition and learning (Bolhuis et al. 1989). The role of predisposition is particularly relevant in the recognition of conspecifics. For example, young Japanese macaques fostered by rhesus macaques exhibit a preference for photos representing members of their own species, even with the lack of experience with conspecific (Fujita 1990, 1993). This is not surprising, as being able to recognize conspecifics quickly and easily is highly beneficial for fitness and it is probable that there has been a strong evolutionary pressure leading to the development of such ability. However, several studies suggest that exposure can, for some species, supersede genetic dispositions. For example, young rhesus macaques raised by Japanese macaques, prefer the pictures of their foster species instead of their own (Fujita 1990). Furthermore, Kendrick et al. (1998) showed that cross-fostered goats and sheep developed a social and a sexual preference towards their foster species which was not altered by later exposure to conspecifics. Exposure seems to be important for the recognition of heterospecifics. For example, domestic companion animals, who are extensively exposed to humans, can discriminate known and unknown humans (Adachi et al. 2007; Proops and McComb 2012; Saito and Shinozuka 2013), and so do captive gorillas living under human care (Salmi et al. 2022) Similarly, exposure provides African forest monkeys with the ability to discriminate between the voices of known and unknown individuals of other monkey species, although the recognition ability is weaker than the one of conspecifics, where there is likely a combined effect of exposure and predisposition (Candiotti et al. 2013). Overall, the literature highlights the complex role of both genetic dispositions and exposure towards species recognition.

Animals’ recognition abilities have been assessed with the use of videos in laboratory settings for several decades (Swartz and Rosenblum 1980; Plimpton et al. 1981), with studies ranging from fish (Gonçalves et al. 2000) to primates (Mosher et al. 2011). Using videos as stimuli offers several advantages: videos convey information about behaviour, which is particularly advantageous when analysing animal’s responses to species-specific features. For instance, videos recordings of female pigeons were more effective than still images in eliciting appropriate social responses from males (Shimizu 1998). Therefore, although the richness of information in videos could also be a source of distraction and/or complexity, it is likely to convey species-specific cues that are crucial to recognition. Compared to live stimuli, videos lack some visual aspects, such as depth cues, making the recognition from videos more difficult (D’Eath 1998). However, as opposed to the exposure to real animals, the use of videos allows for eliminating the presence of olfactory cues, which are scarcely controllable, and therefore to assess the ability of recognition solely based on visual and/or auditory information. Moreover, visual and auditory information can also be detangled in videos making it possible to use cross-modal paradigm. One way to use cross-modal presentations to explore recognition abilities is in expectancy violation paradigms, in which the viewer associates the two sensory cues as belonging to the same class (e.g., species). Using videos with cross-modal paradigm has proved to be a suitable method for assessing recognition abilities in non-human animals (Evans et al. 2005; Adachi and Hampton 2011).

Several studies have looked into the recognition abilities of dogs. These mostly used 2D static stimuli i.e., photographs. For instance, studies have addressed dogs’ ability to recognize conspecifics (Fox 1971; Range et al. 2008; Autier-Dérian et al. 2013; Gergely et al. 2019) and known humans (Adachi et al. 2007; Eatherington et al. 2020). One recent study demonstrated that dogs recognize conspecific from videos (Mongillo et al. 2021). The study employed a cross-modal violation of the expectancy paradigm, pairing videos and vocalizations of a dog and an unfamiliar species. In the context of cross-modal recognition experiments, if the dog shows surprise, classically expressed by a longer looking time, after being presented with an incoherent, but not coherent sequence of stimuli, it should be concluded that the animal had recognized the latter. Such an effect was indeed observed by Mongillo et al. (2021), as dogs looked for a shorter time after being presented with the stimuli if both modalities represented a dog than if one of them represented an unknown species. One question arising from this study was whether dogs showed a different response to the pairing of a bark and a dog video merely because they were familiar with both stimuli, without implying the classification of the stimuli as belonging to a dog (Mongillo et al. 2021). This question encourages the use of videos to assess dogs’ recognition abilities even further, possibly extending to the recognition of other familiar species. In fact, up-to-date studies assessing dogs’ recognition abilities have been limited to conspecifics and humans (Adachi et al. 2007; Mongillo et al. 2021; Ratcliffe et al. 2014). However, assessing the recognition of species to which dogs have had a certain degree of exposure could help shed light on the role of familiarity in recognition abilities. To this aim, cats represent an ideal choice as the species to assess recognition of, as it is relatively easy to find dogs with different levels of exposure to them.

Hence, the aims of the current study were to assess whether dogs can recognise cats as a potentially familiar species and how does continuous exposure influence such abilities. To reach our aim we employed a cross-modal violation of the expectancy paradigm, presenting dogs cohabiting or not cohabiting with cats, with stimuli composed by combinations of a cat or dog video and a cat or dog vocalization. Evidence of increased attention—an indicator of surprise—to the presentation of incoherent combinations (e.g., a meow and a dog’s video), compared to the coherent meow-cat combination, would indicate the ability to recognize cats. Evidence of such surprise reaction in dogs cohabiting with cats, and not in dogs who had never been living with cats, would indicate an important role of regular exposure to cats in adult life towards recognition; vice-versa, lack of recognition in both groups of dogs would indicate that regular exposure is not sufficient to grant recognition abilities. We also expected to observe a surprise reaction towards incoherent combinations over the bark-dog video combination, indicating recognition of conspecifics and replicating the results of our previous study with a similar setup (Mongillo et al. 2021).

## Methods

### Subjects

The sample consisted of sixty-four dog-owner dyads, who were recruited via the database of volunteers at the Laboratory of Applied Ethology at the University of Padua. The recruitment criteria included the dogs being in good health, having no visual or auditory deficits, and being at ease in unfamiliar contexts. Twenty-eight of the dogs were mixed breeds and the remainder were purebreds from various breeds. Their average age ± SD was 5.2 ± 3.3 years, 30 dogs were females, and 34 dogs were males. Half of the recruited dogs were currently co-habiting with cats and the other half of the sample was not currently, neither had previously co-habited with cats. Data regarding the co-habitation with cats was collected by asking the owner if there were one or more cats in the household and if yes, how many of them. If not, the owner was asked if the dog had lived with cats previously. Dogs that were not living with cats currently, but had previously done so, were not included in the experiment.

### Stimuli

Dogs were presented with a combination of a dog or cat vocalization(s) and a dog or cat video, respectively. There were four possible vocalizations, two being recordings of a dog barking, each composed of either 4 or 5 bark bouts, and two being recordings of a cat meowing, each composed of 2 meow bouts. All recordings belonged to different individuals, to exclude possible influences of one particular recording or individual. All four vocalizations had the exact same duration of 2 s and were normalized for intensity at − 6 dB.

There were also four possible visual stimuli consisting of either a dog or a cat walking laterally across the presentation area. Two videos portrayed a dog, one featuring a light-coated mixed breed dog on a black background, while the other video featuring a dark-coated mixed breed dog on a white background. The two videos of cats were analogous to those of the dogs: one featured a light-coated cat on a black background and the other featured a dark-coated cat on a white background. The rationale for using two video recordings of each species was to exclude the influence of one particular coat colour, luminescence or individual. In any case, the portrayed animals entered the presentation area from either the left or right side and made approximately two and a half complete leg cycles before disappearing on the opposite side. All videos had the same duration of 3 s, measured from the first to the last frame in which a part of the animal was visible.

To present both species with real-life size and keep the duration of the stimuli equal among conditions, the measurements of the presentation area varied depending on the species portrayed. The presentation area of a dog video had a height of 150 cm and a width of 120 cm, whereas the dogs in the videos had a height of about 60 cm (height a withers). The background area of a cat video had a height of 100 cm and a width of 80 cm. The portrayed cats had a height of about 25 cm.

Stimuli were paired to obtain four possible combinations: (a) a dog vocalization with a dog video, (b) a dog vocalization with a cat video (c) a cat vocalization with a cat video (d) a cat vocalization with a dog video. Each combination started with the playback of the vocalization and the video started immediately after the end of the vocalization. The animal portrayed in video entered the presentation area from the same side where the vocalization was played, i.e., if the vocalization was played from the right-hand side, the video represented an animal walking from right to left.

### Experimental setting

**Fig. 1:**
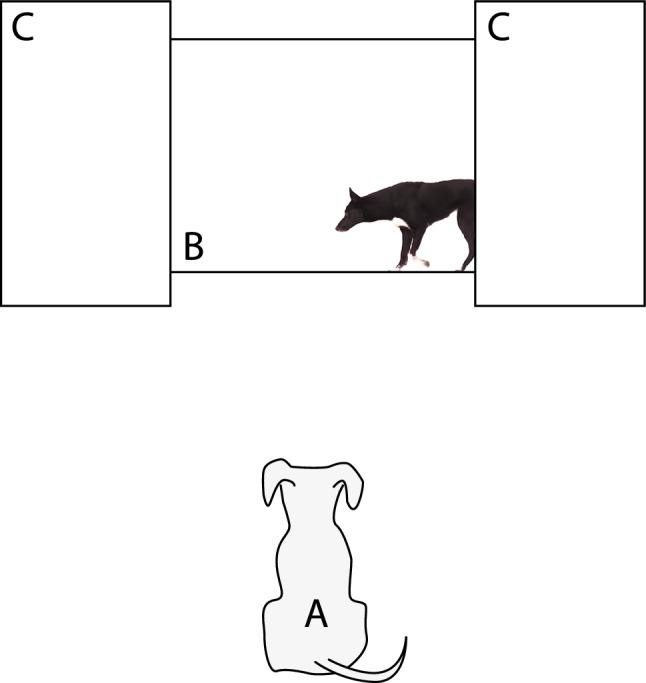
Schematic representation of the experimental setting, illustrating the position of the dog (**A**), the presentation area (**B**) and the two side screens (**C**). Figure elements are not to scale

The experiment was conducted in a quiet room measuring 470 × 580 cm (Fig. 1). A large white plastic screen (152 cm high, and 206 cm wide), on which the stimuli were presented, was placed in the middle of the room. The screen had a hole with a diameter of 6 cm at a height of 96 cm to allow recording the dogs´ orientation from behind the screen (see below). Two smaller screens (152 cm high, and 100 cm wide) were placed about 20 cm in front of the sides of the large one, leaving visible the presentation area to create an impression that the animal presented in the video appeared and disappeared behind the walls (Fig. 1). Two speakers (Hercules XPS 2.0, Hercules Computer Technology, CA, USA) were placed behind the small screens facing the presentation area. A Toshiba TDP T100 projector was mounted at a height of 180 cm on the wall facing the screens. Both the projector and speakers were connected to a MacBook Pro laptop (Apple Computers Inc., Cupertino, CA, USA), which was used to control the presentation of the stimuli by an experimenter, who was sitting behind the screens. During the presentation, dogs were either sitting or standing at a distance of 110 cm from the presentation area, between the legs of their owner who was seated on a small stool behind them.

A CCTV camera was mounted on the ceiling above the dog, recording both the dog and the presentation area during the experiment. A Canon XA20 (Canon, Tokyo, Japan) camcorder was placed behind the screen, pointing via the hole in the screen towards the dog’s face. This camera was set in infrared recording mode which allowed determining the dogs’ eye orientation also in dim light conditions. During the stimuli presentation, the ceiling lights of the experimental room were turned off. To allow video recording, a green spot-on dim light illuminating only the dog was mounted on the ceiling.

### Experimental procedure and design of the experiment

Each dog underwent two trials, in which it was shown a different combination of vocalization and video. Each trial started with the owner leading the dog into the experimental room. Owners were asked to position the dog in a marked location facing the presentation area and gently hold the dog while looking down at their lap to not influence the dog’s behaviour. When the dog was calmly sitting or standing in the predetermined location facing the presentation area, the experimenter started the presentation of the stimuli, which consisted of the playback of the vocalization followed by the video of the animal crossing the presentation area. After the disappearance of the video, the presentation area was set to remain in the background colour (either black or white, depending on the video) for 20 s. During this interval, the owners were instructed to keep looking at their laps and not to interfere with the behaviour of the dog. After the 20 s had passed, the experimenter turned on the lights and asked the owner to leave the room with the dog.

The number of trials each dog underwent was limited to two, as previous research using the same paradigm showed that dogs’ attention towards the presentation area decreases significantly after the second trial (Mongillo et al. 2021). The interval between the two trials was 5 min, in which the owner was asked to wait in a separate room with the dog. Each of the four possible combinations of auditory and visual stimuli was presented 32 times. The four combinations were counterbalanced across the sample in terms of the order in which they were presented (1st or 2nd trial), side of vocalization playback and entrance of the animal portrayed in the video (left or right) and use of black or white background. Some constraints were applied to the two trials to which the same dog was exposed. Specifically, they had to represent a different combination of stimuli (i.e., they could not be both bark + cat), a different side of audio playback and animal entrance (once right and once left), but the same colour combination (either black animal on a white background or vice versa).

### Data collection and analysis

The data regarding the dogs’ head orientation during the experiment was collected and extracted from videos using the Observer XT software (version 12.5, Noldus, Groeningen, The Netherlands). Coding was blind (i.e., the presentation area in the videos were masked and audio was eliminated) so the coder was unaware of the stimulus type projected when coding the dogs’ orientation. Videos were coded using a continuous sampling technique from the appearance of the auditory stimuli until 20 s after the animal had disappeared from the screen. The presentation area was equally divided into three parts and dogs’ head orientation was coded as either looking centrally (towards the central part of the screen) at the entrance (the side where the projected animal came in from), at the exit (the side where the projected animal left from), or elsewhere (looking anywhere else in the room).

The data regarding the dogs’ attention towards the presentation area was collected in two intervals. The first spanning from the onset of the auditory stimulus to the disappearance of the visual stimulus; attention in this interval was associated with the presence of the stimulus. The second interval included the 20 s following the disappearance of the visual stimulus; the dogs’ attention during this interval was thought to reflect a possible surprise effect due to the combination of the audio-visual stimuli. For both intervals, four variables were obtained, respectively, the total time the dog was oriented at the entrance (*looking at the entrance*), centrally (*looking centrally*), at the exit (*looking at the exit*), and at the entire presentation area (i.e., the sum of the previous three variables, *looking at the presentation area*).

To test if the data collected was reliable, an inter-observer reliability was assessed on data coded by a second independent observer. The latter coded data about the dogs’ head orientation on a randomly selected subset of videos (*N* = 32; 25% of the total number). The data collected by the two observers were highly correlated (Looking at the entrance ICC = 0.89; Looking centrally ICC = 0.85; Looking at the exit ICC = 0.86).

For the aim of the analyses, dogs were classified as co-habiting, or not co-habiting with cats. The data were analysed using Generalised Linear Mixed Models (GLM). The first set of analyses was aimed at determining whether dogs’ overall level of attention to the presentation area towards different combinations of auditory and visual stimuli was influenced by the order of trial presentation or the co-habitation with cats. Two models were run, using the time spent looking at the presentation area during and after the stimuli presentation, respectively. In both cases, the model included the dogs’ name as a random factor accounting for repeated measures taken from each dog. Independent variables were the trial order (1st or 2nd trial), the type of stimulus (the four possible combinations of auditory and visual stimulus), the co-habitation with cats (yes or no), an interaction between the type of stimulus and the co-habitation with cats and an interaction between the type of stimulus and the trial order.

The second set of analysis was aimed at assessing dogs’ looking pattern in more detail. In particular, we assessed whether the type of stimulus and the co-habitation with cats influenced dogs’ attention towards different parts of the presentation area, both during and after the stimuli presentation. These analyses are useful to understand specific effects of the individual stimuli (e.g., mainly how much attention is drawn by the video or by the audio), allowing some inference on mechanisms underlying dog’s orientation, beyond the pair’s coherence. To this aim, separate models were run for the variables looking at the entrance, looking centrally or looking at the exit, either during or after the presentation of the stimuli, for a total of six models. The models included the dogs’ name as a random factor. Independent variables were the type of stimulus, co-habitation with cats and their interaction. Trial order was not added as a factor, as the previous models provided evidence that it does not have an effect on the dogs’ attention.

Sequential Bonferroni corrections were applied to all post-hoc pairwise comparisons, as needed. The data were analysed with SPSS (ver. 26; IMB, Armonk, NY). The results are reported as mean ± SD unless otherwise stated.

## Results

For the group of dogs living together with cats, the owners reported that on average there were 2.0 cats present in the household (mode = 1, median = 2).

During the presentation of the stimuli, dogs spent on average 4.6 ± 0.5 s oriented towards the entire presentation area. On average dogs looked for 4.7 ± 0.3 s when presented with coherent dog-related stimuli, for 4.7 ± 0.4 s when presented with a dog video preceded by a cat vocalization, for 4.6 ± 0.3 s when presented with a dog vocalization and a cat video and for 4.5 ± 0.5 s when presented with coherent cat-related stimuli. The model revealed that the interaction between the type of stimulus and the co-habitation with cats had an effect on the dogs’ overall attention, but the pairwise comparisons did not reveal any significant effects (Table 1). No effect of the trial order, the type of stimulus nor the interaction of the latter two was found on such variable.

**Table 1:** Generalized linear mixed model assessing the effect of co-habitation with cats, the trial order, the type of stimulus, an interaction between the co-habitation with cats and type of stimulus and an interaction between the trial order and the type of stimulus on dogs’ looking time at the presentation area during the presentation of stimuli

Factor	Looking at the presentation area	
Co-habitation with cats	*Χ*^*2*^ = 1.52	*p* = 0.22
Trial order	*Χ*^*2*^ = 2.68	*p* = 0.10
Type of stimulus	*Χ*^*2*^ = 1.58	*p* = 0.67
Co-habitation with cats * Type of stimulus	*Χ*^*2*^ = 9.50	*p* = 0.02
Trial order * Type of stimulus	*Χ*^*2*^ = 2.33	*p* = 0.51

**Fig. 2:**
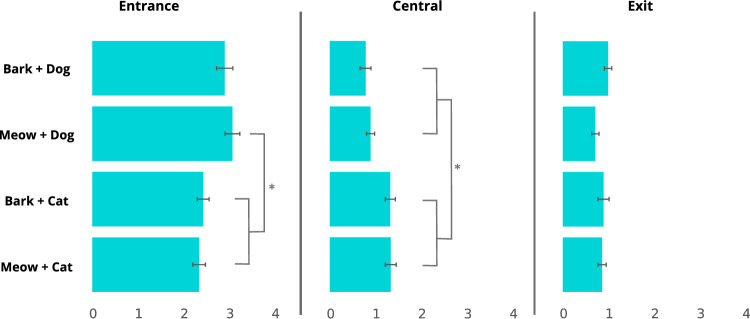
Mean ± SE attention (s) paid at different parts of the presentation area during the stimuli presentation as a function of the type of stimulus (*p < 0.05)

During the presentation of stimuli, dogs spent on average 2.7 ± 0.9 s oriented towards the entrance, 1.1 ± 0.7 s oriented towards the central area, and 0.9 ± 0.5 s oriented towards the exit. The type of stimulus affected the dogs’ looking behaviour towards the entrance (*Χ*^*2*^ = 15.38, *p* = 0.002) and the central area (*Χ*^*2*^ = 18.88, *p* < 0.001), but not towards the exit (*Χ*^*2*^ = 6.4, *p* = 0.09). Specifically, dogs looked longer at the entrance when presented with a meowing dog compared to a meowing cat (*p* = 0.004) or a barking cat (*p* = 0.01) (Fig. 2). Dogs looked less towards the central area when presented with a barking dog compared to a barking cat (*p* = 0.003) or a meowing cat (*p* = 0.007). As well, dogs looked less towards central area when presented with the meowing dog compared to a barking cat (*p* = 0.007) or a meowing cat (*p* = 0.015). The co-habitation with cats (entrance *Χ*^*2*^ = 0.90, *p* = 0.34; central *Χ*^*2*^ = 0.01, *p* = 92; exit* Χ*^*2*^ = 0.18, *p* = 0.67) and the interaction between the latter and the type of stimulus (entrance *Χ*^*2*^ = 3.46, *p* = 0.33; central *Χ*^*2*^ = 5.63, *p* = 0.13; exit* Χ*^*2*^ = 2.41, *p* = 0.49) had no significant effect on the dogs’ looking behaviour in this phase.

**Fig. 3:**
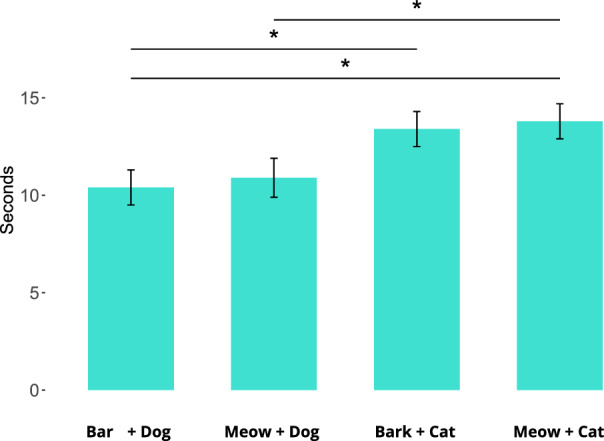
Mean ± SE time (s) spent looking at the presentation area after the stimuli had disappeared, for each of the four types of stimulus (*p < 0.05)

After the stimuli had disappeared, dogs looked at the presentation area for 12.1 ± 5.5 s. On average, dogs looked at the presentation area for 10.3 ± 3.5 s after being presented with coherent dog-related stimuli, for 11.1 ± 3.9 s after being presented with a meowing dog, for 13.1 ± 3.6 s after being presented with a barking cat and for 13.8 ± 3.6 s after being presented with coherent cat-related stimuli. The overall attention paid to the presentation area was affected by the type of stimulus, but not by the other factors (Table 2). The pairwise comparison revealed that dogs looked longer after a cat vocalization was paired with a cat video compared to when a dog video was paired either with a dog vocalization (*p* = 0.01) or a cat vocalization (*p* = 0.01) (Fig. 3). Also, dogs looked longer after a dog vocalization that was paired with a cat video compared to a dog vocalization paired with a dog video (*p* = 0.02).

**Table 2:** Generalized linear mixed model assessing the effect of co-habitation with cats, the trial order, type of stimulus an interaction between the co-habitation with cats and type of stimulus and an interaction between the trial order and the type of stimulus on dogs’ looking time at the presentation area after the presentation of stimuli

Factor	Looking at the presentation area	
Co-habitation with cats	*Χ*^*2*^ = 0.34	*p* = 0.56
Trial order	*Χ*^*2*^ = 1.51	*p* = 0.22
Type of stimulus	*Χ*^*2*^ = 10.50	*p* = 0.02
Co-habitation with cats * Type of stimulus	*Χ*^*2*^ = 1.79	*p* = 0.62
Trial order * Type of stimulus	*Χ*^*2*^ = 0.30	*p* = 0.96

**Fig. 4:**
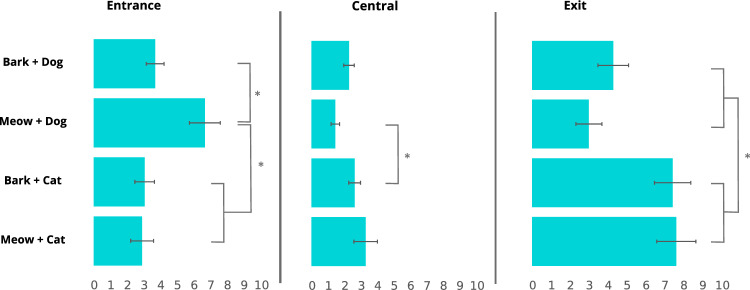
Mean ± SE attention (s) paid at different parts of the presentation area after the stimuli presentation as a function of the type of stimulus (*p < 0.05)

The more detailed analyses of dogs’ looking behaviour after the stimuli presentation showed they spent 4.1 ± 4.3 s oriented towards the entrance, 2.4 ± 2.7 s towards the central area and 5.6 ± 5.4 s towards the exit. The type of stimulus significantly affected attention towards each of the three parts of the presentation area (entrance *Χ*^*2*^ = 13.62, *p* = 0.003; central *Χ*^*2*^ = 12.91, *p* = 0.005;* Χ*^*2*^ = 19.89, exit* p* < 0.001). Dogs looked longer at the entrance after being presented with a meowing dog compared to a barking dog, a barking cat or a meowing cat (Fig. 4). They looked longer at the central area after being presented with a barking cat compared to a meowing dog. Finally, dogs looked longer at the exit after being presented with the combinations containing a cat video compared to the combinations containing a dog video. The co-habitation (entrance *Χ*^*2*^ = 0.11, *p* = 0.74; central *Χ*^*2*^ = 1.07, *p* = 0.30; exit* Χ*^*2*^ = 0.76, *p* = 0.38) or the interaction between the type of stimulus and the co-habitation (entrance *Χ*^*2*^ = 0.87, *p* = 0.83; central *Χ*^*2*^ = 1.38, *p* = 0.71; exit* Χ*^*2*^ = 0.32, *p* = 0.96) did not influence the dogs’ looking behaviour towards any part of the presentation area.

## Discussion

In this study, we assessed if dogs recognize cats in a cross-modal presentation, using a violation of expectancy paradigm and analysing dogs’ attention patterns both during and after the presentation of the stimuli. While exposed to the stimuli, dogs paid the same amount of attention to the area where the stimuli were presented, regardless of what combination of a dog or cat vocalization and a dog or cat video they were exposed to. After the exposure to the stimuli, dogs paid less attention to the presentation area if they had been exposed to a bark-dog pair than a bark-cat pair, but not to that of a meow-dog pair. They also paid more attention to the meow-cat than the meow-dog, but not the bark-cat pair. Thus, attention was largely determined by the video component of the pairs, and yet not exclusively by it, since no difference in attention was found after exposure to the two incoherent pairs, which had different video components. The lack of a surprised reaction, i.e., of an increased attention, to the incoherent stimuli over the meow-cat pair suggests dogs did not recognize cats. However, there was also only limited evidence of a surprised reaction when dogs were exposed to the incoherent stimuli than the bark-dog pair. This result does not fully fall in line with expectations and prompts us to explore factors that might have contributed to driving dogs’ attentional responses in our experiment.

During the presentation of the stimuli, most of the dogs’ attention was drawn by the presence of the stimuli themselves. Dogs remained oriented to the presentation area for most of the duration of the presentation, and regardless of which stimuli were presented. This pattern is unsurprising, considering the suddenness of appearance and salience of the stimuli that crossed the screen in front of the dog. Analogous amounts of attention were observed in a previous study by our laboratory with a similar setup (Mongillo et al. 2021). However, a detailed analysis of the dogs’ orientation revealed they followed more closely the cat video moving across the area than they did with the dog. A likely explanation is that the cat’s video was novel or unusual for the dogs. Dogs’ attraction to novel stimuli has been demonstrated in several other studies (Kaulfuß and Mills 2008; Racca et al. 2010; Lehoczki et al. 2020; Törnqvist et al. 2020, but see also Somppi et al. 2012). Novelty would also explain why, when the stimuli combination featured a dog video, dogs tended to remain oriented towards the entrance region for longer if the preceding vocalization was a meow, compared to a barking (an affect that was apparent in this interval and stronger in the post-exposure phase). What remains to be explained is why such an attractive effect of a novel vocalization did not emerge when the cat videos were shown. One possible explanation is that the two stimuli modalities were not equally salient for dogs, so that the attractive effect of novelty was stronger for the video than for the vocalization and emerged in the latter only when the vocalization was paired with a non-novel stimulus, i.e., the dog video. The greater salience of visual than auditory information for dogs has been reported before, for instance by showing that dogs respond more consistently to visual than vocal discriminatory stimuli (D’Aniello et al. 2016; but see also Gibsone et al. 2021). Beyond an inherent differential salience of the two stimuli, the stimuli presentation sequence might also have impacted the attractiveness of the stimuli, resulting in an increased salience of the one presented as last, i.e., the video. Overall, it seems sensible to assume that novelty acted as an attractor of attention and that its effects were differentially exerted when pertaining to stimuli with different salience. It must be noted that the perception of a stimulus as novel does not necessarily imply a lack of recognition—a stimulus can be recognized but attract attention for being rarely seen. If this was the case, we would have expected to observe a difference in attention between dogs cohabiting with cats and those who did not. However, this was not the case, suggesting that novelty arose from lack of recognition, rather than unusualness of the stimulus. More evidence in this sense comes from the analysis of dogs’ attention after the presentation of the stimuli.

The time interval immediately following exposure to the stimuli was considered more informative about the dog’s expectations, as the patterns of attention would not be directly affected by the presence of stimuli themselves, allowing the eventual effect of a surprise to emerge. It is, therefore, within this interval that we expected to observe higher attention (a manifestation of surprise) after exposure to the incoherent than the coherent stimuli pair, had recognition occurred. The results clearly indicate that this did not happen with the cat-related stimuli. In fact, the coherent meow-cat combination received equal or even higher attention than the incoherent pairs, indicating that the cat stimuli were not recognized as coherent by dogs. Unexpectedly, however, mixed results were also obtained regarding the recognition of the dog stimuli. A surprise reaction was observed when dogs were exposed to the bark-cat combination, compared to the coherent dog pair, supporting the recognition of the dog-related stimuli. However, the same was not true when dogs were exposed to the meow-dog combination. A potential explanation is that, even after the stimuli disappeared, the dogs were still mainly interested in the cat vocalization, a putative effect of novelty as discussed above, not allowing the mismatch with the dog video to result in a surprised reaction. The large attention paid to the entrance region in the post-exposure phase, after being exposed to the cat vocalization, would support this explanation. Another (but not alternative) explanation is that, when the stimuli are presented in sequence rather than simultaneously, the surprise effect can only emerge if an expectation is generated by the first presented stimulus and eventually violated by the second, but not vice-versa. In other words, if the first presented stimulus is not recognized, it would not lead to the formation of an expectation and in turn to no surprise reaction. Regardless of the underlying mechanism, the inability of a novel stimulus to result in a surprise reaction when followed by an incoherent stimulus was observed in previous studies. For example, Kondo et al. (2012) found that crows looked longer after they were shown a known conspecific prior to a playback call of an unknown individual. However, no surprise effect was observed after the crows were first presented with an unknown individual prior to a call of a known conspecific. Similarly, Proops and McComb (2012) showed that horses look longer towards the known person after hearing its voice, but no looking preference was observed after hearing the voice of an unknown person. Therefore, our result can be likely explained by the fact that dogs did not recognize the cat vocalization, and hence no expectation was violated when the dog video appeared.

The last result to be discussed is the lack of influence of cohabitation with cats on recognition, which seems to suggest a limited role of exposure during adult life. One tentative explanation is that the number/variety of individual cats dogs were regularly exposed to was too limited to grant them the ability to recognize cats in our setup. Previous literature suggests that the extent of dogs’ social experience with a variety of individuals influences recognition abilities. For instance, Ratcliffe et al. (2014) found that dogs living together with more than two adult people are better at the audio-visual matching of human gender compared to dogs living with fewer people. The idea that recognition abilities in 2D representations are affected by the extent of exposure is also supported by studies in other species. For instance, chimpanzees exposed to a variety of humans perform better at match-to-sample task with human faces compared to the faces of conspecifics (Martin-Malivel and Okada 2007; Dahl et al. 2013), but the advantage disappears if the number of humans they were exposed to is limited (Martin-Malivel and Okada 2007). Along the same line, house cats are unable to recognize their owner in a cross-modal expectancy violation task, while cats exposed to a large variety of people in a cat-café can do so (Takagi et al. 2019).

Besides the exposure to a variety of representatives of the species, the timing of exposure is also important towards recognition, especially in ontogenetically important periods. For instance, infant Japanese monkeys with limited exposure to humans are unable to recognize them in multi-modal presentations (Adachi et al. 2006), while those that had an extensive experience with several human caretakers were found to have a cross-modal representation of both humans and conspecifics (Adachi et al. 2009). For dogs, it is well known that exposure during the so-called socialization phase (i.e., 3–14 weeks of age; Freedman et al. 1961) is crucial for the ability to recognize their own and other species. Unfortunately, it was impossible to obtain reliable data about this phase of life from owners in our study, most of which were unaware of the conditions in which the dog was raised as a puppy, before adoption. Although they reported no current or past problems in the cohabitation between the dog and the cat(s) in their household, this only indicates that the dog could share spaces with a specific cat, not that they were cat-socialized. Studies assessing the relationship between co-living cats and dogs have found that the two species do not necessarily interact (Menchetti et al. 2020). Therefore, we cannot exclude that dogs in our sample had not been socialized to cats during puppyhood, which in turn would likely have negatively impacted their recognition ability.

## Conclusions

This study indicates that dogs are unable to recognize unfamiliar cats in cross-modal presentations. There was also limited evidence of dogs being able to recognise conspecifics, possibly attributable to methodological choices. The results raise several questions, which will need to be addressed. For instance, as the results highlighted a potential role of both the order of presentation and an inherent difference in the salience of the vocalization and video, disentangling the two effects would shed light on the relevance of auditory and visual stimuli towards recognition. In addition, it would be interesting to explore whether recognition abilities would be differently affected by the presentation of sets of stimuli with different informative content, which may include a variety of both context-specific vocalizations and behavioural displays. Moreover, dogs’ inability to recognize cats extended to dogs living with cats, suggesting that exposure to a limited number of individuals, much as regular, is not enough to grant dogs with recognition abilities. This result does not exclude that experience plays an important role in dogs’ recognition abilities, but raises questions about the role of the extent and variety as well as the timing of exposure. To clarify these aspects would certainly require accurate and detailed information about the timing and extent of dogs’ exposure to other species and the possibility of systematically manipulate such variables. It should be noted that these conditions might not be easily achievable in pet dogs and further experiments might require the involvement of experimental animals, whose social experience with other species could be both better known and manipulated.

## Supplementary Information

Below is the link to the electronic supplementary material.

Supplementary file1 (DOCX 24 KB)

## Data Availability

Data are publicly available in the data repository of the University of Padua.
