# Supplementary material for: Meowing dogs: can dogs recognize cats in a cross-modal violation of expectancy task (Canis familiaris)?
Source: Anim Cogn. 2023 May 12;26(4):1335–44. doi: 10.1007/s10071-023-01783-0 (PMC10345037; doi:10.1007/s10071-023-01783-0)
Supplement: Supplementary file 1 — Supplementary file1 (DOCX 24 KB) [file 10071_2023_1783_MOESM1_ESM.docx]

| **Subject nr** | **Sex** | **Age (y)** | **Breed** | **Co-habitation with cats** | **Number of cats present** |
| --- | --- | --- | --- | --- | --- |
| 1 | M | 9.5 | Maltese | Yes | 2 |
| 2 | M | 5.1 | Maltese | Yes | 2 |
| 3 | M | 4.2 | Australian Shepherd | Yes | 2 |
| 4 | M | 13.3 | Mixed breed | Yes | 1 |
| 5 | F | 3.1 | Mixed breed | Yes | 2 |
| 6 | M | 2.2 | Mixed breed | Yes | 5 |
| 7 | F | 8.2 | Labrador Retriever | Yes | 1 |
| 8 | F | 6.4 | Czechoslovakian Wolfdog | Yes | 2 |
| 9 | F | 1.5 | Border Collie | Yes | 1 |
| 10 | M | 3.4 | French Bulldog | Yes | 1 |
| 11 | M | 5.3 | Beagle | Yes | 1 |
| 12 | F | 2.9 | Mixed breed | Yes | 1 |
| 13 | F | 11.5 | Mixed breed | Yes | 1 |
| 14 | F | 9.9 | Czechoslovakian Wolfdog | Yes | 3 |
| 15 | F | 3.6 | Golden Retriever | Yes | 3 |
| 16 | M | 0.9 | Golden Retriever | Yes | 3 |
| 17 | F | 4.9 | Mixed breed | Yes | 4 |
| 18 | M | 1.2 | Mixed breed | Yes | 4 |
| 19 | M | 9.3 | Spanish Greyhound | Yes | 2 |
| 20 | F | 1.0 | Mixed breed | Yes | 1 |
| 21 | M | 7.6 | Mixed breed | Yes | 1 |
| 22 | F | 10.2 | Mixed breed | Yes | 2 |
| 23 | M | 10.2 | Mixed breed | Yes | 2 |
| 24 | F | 2.3 | Siberian Husky | Yes | 2 |
| 25 | F | 3.3 | Rottweiler | Yes | 3 |
| 26 | F | 8.4 | Whippet | Yes | 1 |
| 27 | F | 6.8 | Mixed breed | Yes | 1 |
| 28 | F | 3.1 | Dachshund | Yes | 2 |
| 29 | M | 6.0 | Mixed breed | Yes | 2 |
| 30 | M | 2.2 | Dalmatian | Yes | 1 |
| 31 | M | 8.9 | Labrador Retriever | Yes | 3 |
| 32 | M | 4.0 | Dalmatian | Yes | 1 |
| 33 | F | 3.2 | Pastore del Lagorai | No | - |
| 34 | M | 1.7 | Mixed breed | No | - |
| 35 | M | 8.7 | Mixed breed | No | - |
| 36 | M | 6.8 | Australian Shepherd | No | - |
| 37 | F | 1.0 | Labrador Retriever | No | - |
| 38 | F | 1.3 | Mixed breed | No | - |
| 39 | F | 1.4 | Golden Retriever | No | - |
| 40 | F | 4.3 | Mixed breed | No | - |
| 41 | M | 4.2 | Mixed breed | No | - |
| 42 | M | 2.4 | Amstaff | No | - |
| 43 | F | 1.4 | Shiba Inu | No | - |
| 44 | M | 8.9 | Rhodesian Ridgeback | No | - |
| 45 | M | 5.8 | Mixed breed | No | - |
| 46 | M | 2.1 | Weimaraner | No | - |
| 47 | M | 7.8 | Mixed breed | No | - |
| 48 | F | 4.9 | Boxer | No | - |
| 49 | F | 11.3 | Golden Retriever | No | - |
| 50 | F | 7.1 | Mixed breed | No | - |
| 51 | M | 0.8 | Golden Retriever | No | - |
| 52 | F | 7.6 | Mixed breed | No | - |
| 53 | M | 2.0 | Mixed breed | No | - |
| 54 | M | 6.3 | Mixed breed | No | - |
| 55 | M | 9.7 | Mixed breed | No | - |
| 56 | F | 7.3 | Boxer | No | - |
| 57 | F | 5.4 | Bernese Mountain Dog | No | - |
| 58 | M | 1.0 | Australian Shepherd | No | - |
| 59 | M | 5.1 | Golden Retriever | No | - |
| 60 | F | 3.4 | German Shepherd | No | - |
| 61 | F | 7.0 | Australian Shepherd | No | - |
| 62 | M | 0.7 | Mixed breed | No | - |
| 63 | M | 2.5 | Mixed breed | No | - |
| 64 | M | 10.2 | Mixed breed | No | - |
